# Low Paraoxonase 1 Activity Predicts Mortality in Surgical Patients with Sepsis

**DOI:** 10.1155/2014/427378

**Published:** 2014-02-09

**Authors:** Suzana Bojic, Jelena Kotur-Stevuljevic, Nevena Kalezic, Zorana Jelic-Ivanovic, Aleksandra Stefanovic, Ivan Palibrk, Lidija Memon, Zdravko Kalaba, Marina Stojanovic, Sanja Simic-Ogrizovic

**Affiliations:** ^1^Department of Anaesthesiology, Resuscitation and Intensive Care, Clinical Hospital Center Bezanijska Kosa, Bezanijska kosa bb, 11000 Belgrade, Serbia; ^2^Faculty of Pharmacy, Vojvode Stepe 450, 11000 Belgrade, Serbia; ^3^Clinical Center of Serbia, Pasterova 2, 11000 Belgrade, Serbia; ^4^School of Medicine, University of Belgrade, Dr. Subotica 8, 11000 Belgrade, Serbia; ^5^Clinical Chemistry Laboratory, Clinical Hospital Center Bezanijska Kosa, Bezanijska kosa bb, 11000 Belgrade, Serbia

## Abstract

*Introduction*. State of severe oxidative stress is encountered in sepsis. Paraoxonase 1 (PON1) protects against oxidative stress but also undergoes inactivation upon that condition. We investigated PON1 activity in surgical patients with sepsis in relation to oxidative stress status, inflammation, disease severity, and survival. *Methods*. Prospective observational study. Sixty-nine surgical patients with sepsis were compared to 69 age/sex matched healthy controls. PON1 paraoxonase and diazoxonase activities, selected biochemical, hematological and oxidative stress parameters were measured on admission to ICU and 24, 48, 72, and 96 hours later. Disease severity scores were calculated daily. *Results*. Septic patients had significantly lower PON1 activities compared to control group at all time points. PON1 activities had good capacity to differentiate septic patients from healthy controls. Low PON1 activities were associated with higher disease severity scores and higher risk of death. Correlation between PON1 activity and markers of inflammation failed to reach significance. Decrease in PON1 activity was correlated with an increase in reducing components in plasma. *Conclusion*. Our study demonstrated lower PON1 activity in surgical patients with sepsis compared to healthy controls. PON1 activity also reflected severity of the disease. Low PON1 activity was associated with higher mortality of surgical patients with sepsis.

## 1. Introduction

Immune response in sepsis increases production of reactive oxygen and nitrogen species [1]. If production of these reactive species exceeds organism's ability to detoxify the reactive intermediates or to repair the resulting damage, a state of oxidative stress occurs. A number of studies describe oxidative stress in patients with sepsis with evidence of depleted antioxidant defense [2].

Paraoxonase 1 (PON1) is a calcium-dependent esterase mainly synthesized by the liver [3]. PON1 peroxidase and esterase activities seem to be of major importance in detoxifying oxidative stress mediators which probably explains the antioxidant and anti-inflammatory potential of the enzyme [4]. Growing interest in the enzyme's importance is prompted by its role in lipid peroxidation and the development of atherosclerosis [5]. Decreased PON1 activity was also observed in liver disease [6, 7], acute pancreatitis [8], diabetes [9], chronic renal failure, and dialysis [10, 11]. So far, no data have been reported on the utility of serial PON1 measurement in surgical patients with sepsis.

The objective of this study was to investigate PON1 activity in surgical patients with sepsis in relation to disease severity, oxidative stress status, inflammation, and survival.

## 2. Materials and Methods

### 2.1. Patients

This prospective, observational study included 69 patients (36 men/33 women, age 62.0 (54.5–74.0) years) admitted to university hospital surgical intensive care unit (ICU) for treatment of sepsis during 2011. Sepsis was diagnosed according to American College of Chest Physicians/Society of Critical Care Medicine Consensus Conference criteria [12]. Patients with chemotherapy and radiotherapy in the past 30 days, immunosuppressant therapy, major trauma, end-stage organ disease, cardiogenic, or hemorrhagic shock were excluded. Acute Physiology and Chronic Health Evaluation II score (APACHE II) [13] and Sequential Organ Failure Assessment score (SOFA) [14] were calculated daily. Data regarding mechanical ventilation, inotropic and/or vasopressor support, and survival were obtained from medical records.

Control group consisted of 69 age and sex-matched healthy patients (36 men/33 women, age 61 (54.5–69.0) years), who attended annual medical checkups at health centers in Belgrade, were free of known cardiac, renal, and hepatic diseases and were not taking any prescribed medication. There was no statistically significant difference in age and gender between these groups. The study was approved by institutional ethical committee and written informed consent obtained from participants or their legal representatives.

### 2.2. Samples

Venous blood samples from patients with sepsis were collected during the first hour following admission to ICU (0 h) and 24 h, 48 h, 72 h, and 96 h later. Samples from control group were collected in the morning, after fasting for the night. Blood was drawn into standard collection tubes with cloth activator. Centrifuged serum aliquots used for measurement of PON1 and oxidative stress parameters were frozen to −20°C and stored to −80°C until analysis. Other parameters were analyzed on the day of collection. Lactate levels were measured in arterial blood samples.

### 2.3. PON1 Activities and Oxidative Stress Parameters

Serum paraoxonase-1 (PON1) activity was measured as rate of paraoxon (POase) and diazoxone (DZOase) hydrolysis according to Richter and Furlong [15]. Malondialdehyde (MDA), a marker of lipid peroxidation, was measured using thiobarbituric acid reactive substances method [16]. A colorimetric assay based on the oxidation of ferrous ion to ferric ion in the presence of various oxidant species in acidic medium was used for total oxidant status (TOS) measurement [17]. Total antioxidant status (TAS) was determined with novel automated colorimetric method developed by Erel [18]. The assay used for measurement of prooxidant-antioxidant balance (PAB) is based on 3,3′,5,5′-tetramethylbenzidine and its cation used as a redox indicator participating in two simultaneous reactions [19].

### 2.4. Biomarkers of Inflammation and Infection

White blood cells (WBC) count was measured in hematology analyzers (ABX Horiba, Pentra DX 120, Montpellier, France and Beckman Coulter, AcT diff, Germany). Immunoturbidimetric assay (bioMerieux, Lion, France on the IL 650 analyzer, Milan, Italy) was employed for C reactive protein (CRP) concentration measurement. Serum procalcitonin (PCT) was measured with ELFA method (bioMerieux, Lion, France). With this method, a concentration >2 *μ*g/L represents high risk of severe sepsis and/or septic shock.

### 2.5. Biochemical Parameters

Serum urea, creatinine, and total bilirubin concentrations; aspartate-aminotransferase (AST); and alanine-aminotransferase (ALT) activities were analyzed employing routine methods (Instrumentation Laboratory reagents using the analyzer IL 650, Milan, Italy). Lactate level was measured by blood gas analyzer (GEM Premier 3000, Instrumentation Laboratory, Milan, Italy).

### 2.6. Statistical Analysis

Statistical analysis was performed in SPSS 15.0 software (SPSS Inc., Chicago, IL, USA). Normality of data was assessed with Shapiro-Wilk test. Data were presented as median and 25th to 75th quartile. Friedman test was used to calculate significance of difference in multiple related samples and Mann-Whitney *U* test in nonrelated samples. To determine possible correlation between variables in patients with sepsis, Kendall tau-b test was employed. Areas under receiver operating characteristic curves (AUC-ROC) were also calculated. Kaplan-Meier survival analysis and stepwise multiple linear regression analysis were performed. The minimal statistical significance was set at two-tailed *P* < 0.05.

## 3. Results

On admission to ICU, 36 of 69 patients had uncomplicated sepsis, 23 severe sepsis and 10 were in septic shock. General characteristics, PON1 activities, oxidative stress, and biochemical parameters of the study groups were listed in Table 1. Septic patients had significantly lower POase and DZOase activities compared to control group at all time points. PON1 activities did not changed significantly during first five days in ICU (Friedman *p*_POase_ = 0.302, *p*_DZOase_ = 0.982). State of exacerbated oxidative stress was evidenced through significantly higher TOS, PAB, and MDA levels and lower TAS levels compared to the control group. Oxidative stress parameters in patients with sepsis also did not change significantly for the duration of the study (Friedman *p*_TAS_ = 0.275, *p*_TOS_ = 0.507, *p*_PAB_ = 0.598, and *p*_MDA_ = 0.683).

**Figure 1:**
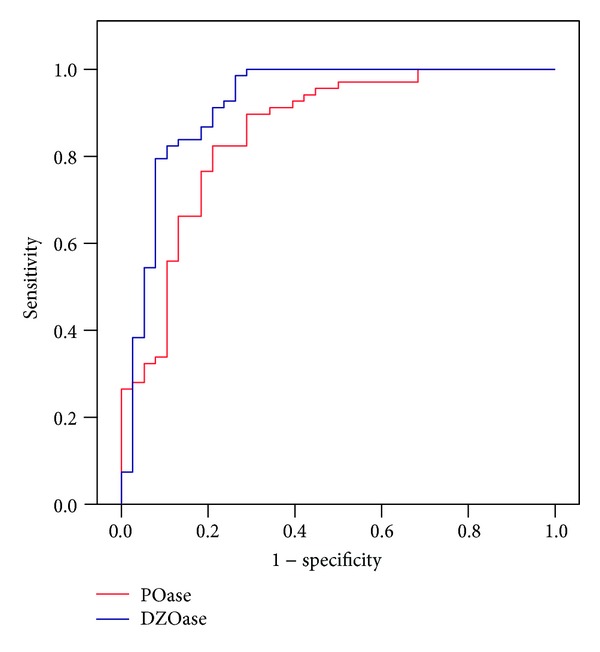
Relevance of POase and DZOase activity on admission to ICU as biomarkers of sepsis. AUC-ROC_POase_ 0.856 (95% CI: 0.777–0.934) *P* < 0.001, AUC-ROC_DZOase_ 0.921 (95% CI: 0.857–0.984) *P* < 0.001. AUC-ROC (area under the receiver operating characteristic curve). Red line-POase; blue line-DZOase.

AUC-ROC was used to evaluate PON1 activities on admission to ICU as a marker of sepsis (Figure 1). AUC-ROC for POase was 0.856 (95% CI: 0.777 to 0.934), *P* < 0.001 and 0.921 (95% CI: 0.857 to 0.984), *P* < 0.001 for DZOase. PON1 activities on admission to ICU had good to excellent capacity to differentiate surgical patients with sepsis from healthy controls.

We found significant positive correlation between POase and DZOase PON1 activities (Table 2). Both POase and DZOase activity positively correlated with calcium concentration (*τ* = 0.271, *P* < 0.01 and *τ* = 0.237, *P* < 0.05, resp.). A trend towards negative correlation between PON1 activities and markers of inflammation and infection was noted but failed to reach significance. PON1 activity and disease severity scores were inversely correlated. In fact, multiple linear regression analysis revealed that SOFA score was independent predictor of both POase and DZOase activity (standardized coefficient *β* = −0.328; *P* = 0.012 and *β* = −0.317; *P* = 0.034, resp.). POase activity correlated negatively with lactate levels but not with markers of renal and liver function.

Unexpectedly, POase activity correlated positively with TOS and PAB. These parameters were included in stepwise multiple linear regression analysis and PAB proved to be a positive predictor of POase activity (standardized coefficient *β* = 0.304; *P* = 0.004). MDA, a marker of oxidative stress, correlated positively with markers of inflammation, infection, and renal and liver function as well as disease severity scores. Surprisingly, it correlated positively with TAS (*τ* = 0.172, *P* < 0.01) and negatively with PAB (*τ* = −0.226, *P* < 0.01). TAS correlated positively and TOS and PAB negatively with PCT values, markers of renal and liver function, and disease severity scores. Further analysis included serum creatinine, bilirubin, and PCT levels as potential predictors of oxidative stress parameters. We found that creatinine and bilirubin levels were significant predictors of MDA concentration (standardized coefficients *β*_creatinine_ = 0.394, *P* < 0.001 and *β*_bilirubin_ = 0.469, *P* < 0.001). Serum creatinine was the only predictor of TAS (standardized coefficients *β*_creatinine_ = 0.401, *P* < 0.001). All three parameters were negative predictors of PAB (standardized coefficients *β*_creatinine_ = −0.362, *P* < 0.001; *β*_PCT_ = −0.284, *P* = 0.004 and *β*_bilirubin_ = −0.189, *P* = 0.040). These parameters were not good predictors of TOS, but after substituting creatinine with urea in this model, we found that urea and PCT levels could predict TOS (standardized coefficients *β*_urea_ = −0.451, *P* < 0.001; *β*_PCT_ = −0.239, *P* = 0.017).

**Figure 2:**
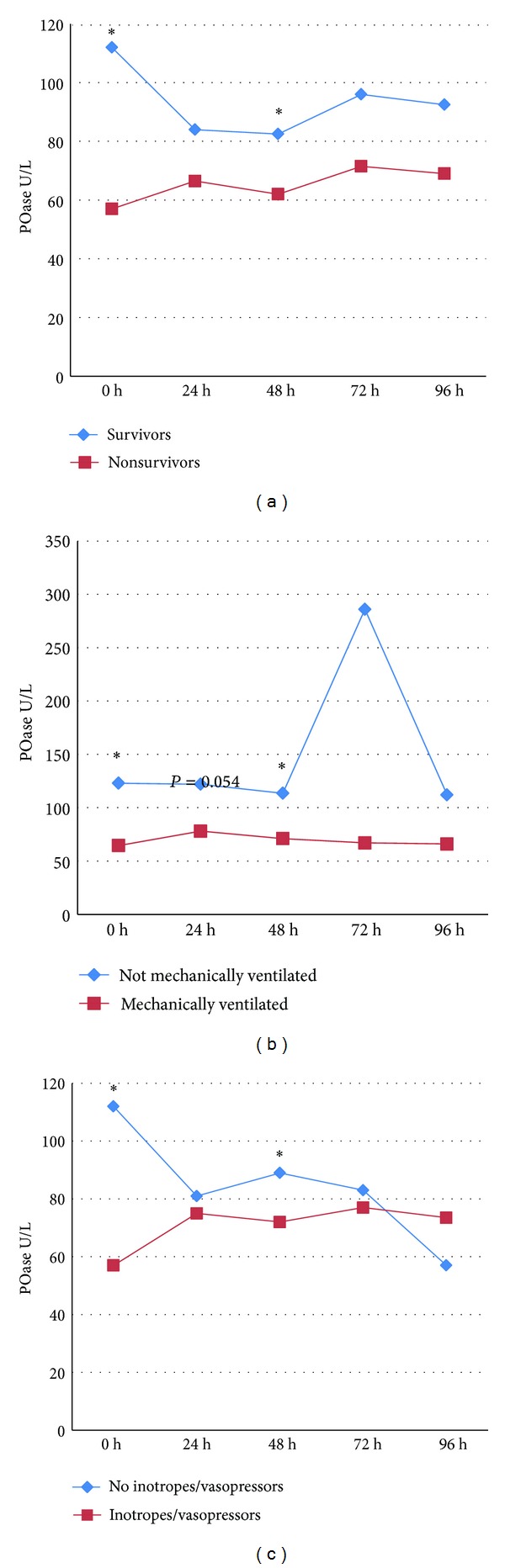
POase activities in patients with sepsis based on survival, mechanical ventilation, use of inotropes, and/or vasopressors. (a) Survivors versus nonsurvivors; (b) mechanically ventilated versus not mechanically ventilated; (c) inotropes and/or vasopressors versus no inotropes and/or vasopressors. Data are presented as medians. *P < 0.05—Mann-Whitney *U* test.

Twenty-six of 69 patients died while treated for sepsis, 37 required mechanical ventilation and 26 inotropic and/or vasopressor support.  Figure 2 presents PON1 activities in patients with sepsis based on survival, mechanical ventilation, and use of inotropic and/or vasopressors, RRT. We observed a trend towards lower POase and DZOase activity in nonsurvivors, mechanically ventilated patients, and in patients requiring inotropic and/or vasopressors. This difference reached statistical significance on admission to ICU and 48 h later. DZOase activities were not statistically different between these groups of septic patients.

**Figure 3:**
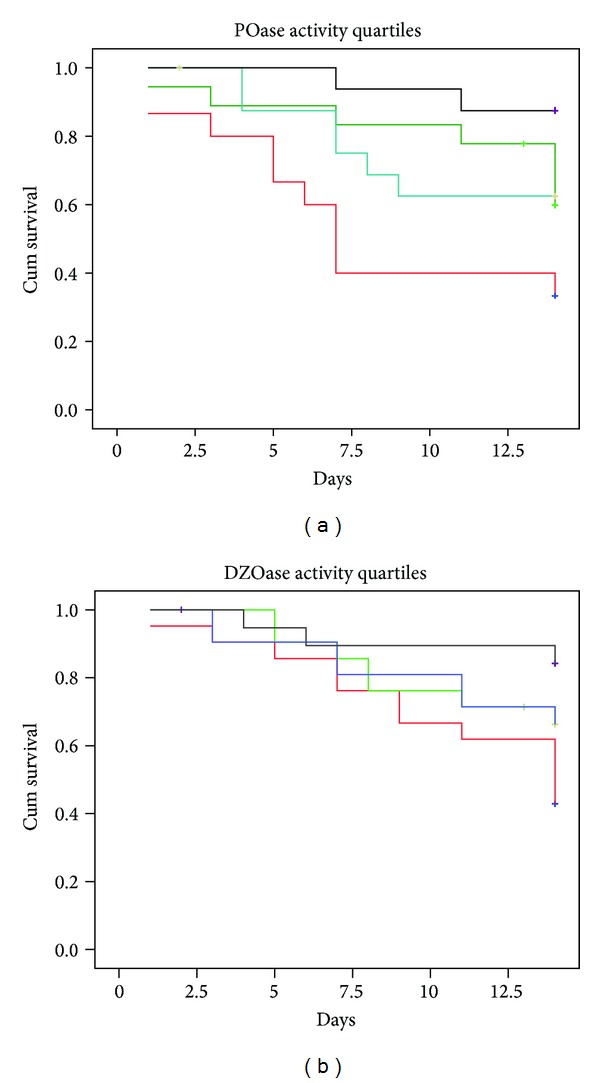
Kaplan-Meier estimates of 14-day survival in patients stratified in (a) POase activity quartiles and (b) DZOase activity quartiles. Log rank: (a) POase *P* < 0.001 and (b) DZOase *P* = 0.075. Black line—4th quartile; blue line—3rd quartile; green line—2nd quartile; red line—1st quartile.

Kaplan-Meier survival analysis presented in Figure 3 clearly shows higher risk of death with lower POase activity (log rank *P* < 0.001), while DZOase activity did not seem to be a good predictor of sepsis outcome (log rank *P* = 0.075).

Surprisingly, higher TAS and MDA values as well as lower TOS and PAB values also implicated higher risk of death (log rank *p*_TAS_ = 0.016, *p*_MDA_ = 0.002, *p*_TOS_ = 0.002, and *p*_PAB_ < 0.001).

## 4. Discussion

Despite considerable interest in the research of PON1 activity in various acute and chronic diseases, importance of PON1 in critical illness is just starting to be explored. Serum PON1 has been demonstrated in multiple clinical and animal studies to protect against oxidative stress but also to undergo inactivation upon that condition [4, 5]. Previous studies showed low PON1 activity in small groups of medical patients with sepsis compared to healthy controls [20–22]. We have also found that both POase and DZOase activity were lower in surgical patients with sepsis than in healthy controls. In the study of Sans et al., POase activity significantly decreased between admission to ICU and the following day but remained unchanged for the next 4 days [21]. In our study, low PON1 activity did not significantly change during first 5 days of disease. PON1 activity also reflected severity of the disease. In our study PON1 activities were lower in patients with higher APACHE II and SOFA scores. Our data are in contrast with results from Novak et al. who did not find statistically significant correlation between PON1 activities and disease severity scores [20], while Sans et al. found this correlation to be significant only after recovery from sepsis [21].

A ROC curve analysis showed both POase and DZOase activities to be good to excellent markers of sepsis. To date, similar evaluation of POase and DZOase activities as diagnostic tests for sepsis was not performed. However, measurement of PON1 arylesterase activity was found to be an efficient test for identifying the presence and severity of chronic liver injury [6].

In this study, septic patients had increased prooxidant and decreased antioxidant status compared to control group implicating state of severe oxidative stress. Surprisingly, higher disease severity scores followed by decrease in PON1 activity were associated with better antioxidant and worse prooxidant status. Chuang et al. also demonstrated positive correlation between total antioxidant capacity and APACHE II score [23]. A possible explanation for this phenomenon could be found in relationship between these parameters and serum creatinine and bilirubin, known reducing type antioxidants [24, 25]. In our study, TAS and MDA correlated positively and TOS and PAB negatively with serum creatinine and bilirubin levels. Moreover, creatinine and bilirubin levels could actually predict oxidative status parameters values. One could argue that increase in creatinine and bilirubin concentrations, caused by renal and hepatic dysfunction, is at least partially responsible for increase in antioxidant and, consequently, decrease in prooxidant status measurement. At the same time, comparison to healthy controls clearly shows that septic patients actually were in a state of severe oxidative stress and had impaired antioxidant defense.

We found no significant correlation between markers of inflammation and PON1 activity. Various research groups have demonstrated that inflammation modulates PON1 activity [5]. Association between CPR and PON1 values in sepsis was observed by Novak et al. [20] but not by Sans et al. [21].

In our study, serum PON1 activities were significantly lower in nonsurvivors than in survivors. Furthermore, lower PON1 activities were associated with higher mortality as previously implicated by Draganov et al. [22]. On the other hand, Novak et al. found no significant difference between survivors and nonsurvivors, but a trend toward lower arylesterase PON1 activity in nonsurvivors as compared to survivors was noticed [20]. Low PON1 activity is also associated with breast cancer mortality [26] and certain genotypes of PON1 gene with lung carcinoma [27]. Lower PON1 activities were also observed in patient requiring mechanical ventilation or inotropes and/or vasopressors. So far, no studies were performed to explore association between these measures of vital support and PON1 activity.

Relatively small and diverse group of patients was the biggest drawback of our study. Further studies are needed to fully investigate role of PON1 in pathogenesis of sepsis.

## 5. Conclusion

This study demonstrated significantly lower PON1 activity in surgical patients with sepsis than in healthy controls. Low PON1 activity was associated with higher disease severity scores and higher risk of death. Decrease in PON1 activity was associated with better antioxidant and worse prooxidant status. Correlation between PON1 activity and markers of inflammation was not observed.

## Figures and Tables

**Table 1:** General characteristics, PON1 activities, oxidative stress, inflammation, and biochemical parameters on admission to ICU (0 h), 24 h, 48 h, 72 h, and 96 h later.

	Controls	Sepsis 0 h	Sepsis 24 h	Sepsis 48 h	Sepsis 72 h	Sepsis 96 h
Age (years)	61.0 [54.5–69.0]	62.0 [54.5–74.0]	—	—	—	—
Gender (m/f)	36/33	36/33	—	—	—	—
APACHE II	—	23 [19–35]	20 [14–28]	23 [13–31]	23 [11–28]	25 [15–30]
SOFA	—	7 [3–14]	6 [2–8]	6 [0–11]	8 [4–14]	8 [2–13]
POase (U/L)	321.8 [159.7–548.6]	89.0 [38.0–140.0]^a^	78.0 [55.0–117.0]^a^	76.0 [50.5–114.5]^a^	77.0 [41.0–119.0]^a^	69.0 [48.5–118.0]^a^
DZOase (U/L)	10822.9 [785.4–14613.7]	3263.0 [2496.5–4981.5]^a^	3037.0 [2754.5–6829.5]^a^	3046.0 [2378.0–4467.0]^a^	3058.0 [3016.0–4130.5]^a^	5737.0 [4871.5–7158.5]^a^
TAS (*μ*mol/L)	1114.2 [1032.8–1212.2]	714.0 [532.0–954.0]^a^	899.0 [597.0–988.0]^a^	696.0 [588.0–963.0]^a^	984.0 [621.0–1076.0]^a^	1049.0 [752.9–1071.0]^a^
TOS (*μ*mol/L)	4.65 [3.60–5.40]	6.05 [4.00–11.50]^a^	5.35 [3.00–10.10]	5.80 [2.80–12.00]	7.20 [3.60–8.80]^a^	4.20 [3.10–7.25]
PAB (HKU)	14.3 [11.5–15.6]	23.7 [10.0–83.3]^a^	13.1 [5.9–59.4]	42.7 [11.0–91.9]^a^	12.2 [4.4–48.5]	12.8 [5.7–74.0]
MDA (*μ*mol/L)	0.85 [0.41–1.11]	1.09 [0.70–1.52]^a^	1.91 [1.13–2.85]^a^	1.05 [0.83–1.38]^a^	1.42 [1.07–2.64]^a^	1.99 [1.11–2.42]^a^
WBC × 10^9^/L	—	15.9 [10.3–20.4]	10.8 [8.3–16.9]	12.4 [8.5–17.7]	13.3 [8.1–16.3]	14.6 [9.0–21.7]
CRP (mg/L)	—	172.1 [78.3–226.1]	141.9 [97.8–204.0]	136.6 [102.9–193.4]	93.0 [87.5–150.1]	61.2 [55.2–161]
PCT (*μ*g/L)	—	3.9 [1.6–14.0]	9.8 [4.5–31.2]	3.7 [1.3–8.6]	28.2 [6.1–48.4]	9.2 [4.5–16.8]
Urea (mmo/L)	—	10.6 [5.7–18.9]	13.5 [9.1–21.1]	11.5 [4.3–18.7]	19.4 [12.3–27.5]	24.2 [14.2–28.7]
Creatinine (*μ*mol/L)	—	112.5 [91.0–168.1]	136.7 [80.9–176.8]	107.4 [75.4–156.3]	129.1 [82.1–259.7]	188.2 [105.0–275.2]
Bilirubin (*μ*mol/L)	—	14.6 [7.8–22.3]	24.5 [13.1–72.7]	10.4 [8.2–19.1]	20.7 [13.7–48.0]	18.4 [9.2–56.5]
AST (IU/L)	—	28 [16–76]	57 [29–88]	25 [14–42]	24 [19–116]	26 [21–111]
ALT (IU/L)	—	23 [12–59]	38 [19–79]	18 [13–37]	29 [23–76]	26 [17–82]
Lactate (mmol/L)	—	1.3 [1.0–3.2]	1.5 [0.9–2.2]	1.5 [1.2–3.3]	1.8 [1.1–2.6]	1.3 [1.2–3.6]

Data are presented as median and 25th–75th percentile values. ^a^Compared to control group, *P* < 0.05—Mann-Whitney *U* test. APACHE II: Acute Physiology and Chronic Health Evaluation II score; SOFA: Sequential Organ Failure Assessment score; TAS: total antioxidant status; TOS: total oxidant status; PAB: prooxidant-antioxidant balance; MDA: malondialdehyde; WBC: white blood cells; CRP: C reactive protein; PCT: procalcitonin (PCT); AST: aspartate-aminotransferase; ALT: alanine-aminotransferase.

**Table 2:** Correlation coefficients between listed parameters in surgical patients with sepsis.

Kendal *τ*_*B*_	POase	DZOase	TAS	TOS	PAB	MDA
POase	1.000	0.243**	−0.033	0.160**	0.140**	−0.104
DZOase	0.243**	1.000	−0.034	0.080	0.020	−0.026
WBC	−0.056	−0.063	0.008	−0.004	−0.016	−0.071
CRP	−0.065	−0.098	0.022	−0.004	0.025	0.171*
PCT	0.037	0.043	0.227**	−0.022	−0.350**	0.182*
APACHE II	−0.185**	−0.081	0.235**	−0.191**	−0.402**	0.182**
SOFA	−0.111	−0.232*	0.176*	−0.127	−0.422**	0.189*
Urea	−0.096	−0.107	0.309**	0.209**	−0.401**	0.234**
Creatinine	−0.077	−0.017	0.319**	−0.174**	−0.419**	0.202**
Bilirubin	0.020	−0.013	0.176*	−0.047	−0.241**	0.439**
AST	0.098	0.107	0.188**	−0.011	−0.314**	0.323**
ALT	0.202**	0.096	0.163*	0.062	−0.136*	0.256**
Lactate	−0.179**	−0.049	0.118	−0.010	−0.205**	0.125

*P < 0.05 (2-tailed); **P < 0.01 (2-tailed). Kendal tau b correlation analysis. TAS: total antioxidant status; TOS: total oxidant status; PAB: prooxidant-antioxidant balance; MDA: malondialdehyde; APACHE II: Acute Physiology and Chronic Health Evaluation II score; SOFA: Sequential Organ Failure Assessment score; WBC: white blood cells; CRP: C reactive protein; PCT: procalcitonin (PCT); AST: aspartate-aminotransferase; ALT: alanine-aminotransferase.
